# Performance of a fully-automated Lumipulse plasma phospho-tau181 assay for Alzheimer’s disease

**DOI:** 10.1186/s13195-022-01116-2

**Published:** 2022-11-12

**Authors:** Edward N. Wilson, Christina B. Young, Javier Ramos Benitez, Michelle S. Swarovski, Igor Feinstein, Manu Vandijck, Yann Le Guen, Nandita M. Kasireddy, Marian Shahid, Nicole K. Corso, Qian Wang, Gabriel Kennedy, Alexandra N. Trelle, Betty Lind, Divya Channappa, Malia Belnap, Veronica Ramirez, Irina Skylar-Scott, Kyan Younes, Maya V. Yutsis, Nathalie Le Bastard, Joseph F. Quinn, Christopher H. van Dyck, Angus Nairn, Carolyn A. Fredericks, Lu Tian, Geoffrey A. Kerchner, Thomas J. Montine, Sharon J. Sha, Guido Davidzon, Victor W. Henderson, Frank M. Longo, Michael D. Greicius, Anthony D. Wagner, Tony Wyss-Coray, Kathleen L. Poston, Elizabeth C. Mormino, Katrin I. Andreasson

**Affiliations:** 1 Neurology & Neurological Sciences, Stanford University, Stanford, CA USA; 2 Wu Tsai Neurosciences Institute, Stanford University, Stanford, CA USA; 3 Anesthesiology, Perioperative and Pain Medicine, Stanford University, Stanford, CA USA; 4 Fujirebio Europe NV, Ghent, Belgium; 5 Psychology, Stanford University, Stanford, CA USA; 6 Neurology, Portland VA Medical Center, Portland, OR USA; 7 Neurology, Oregon Health & Science University, Portland, OR USA; 8 Pathology, Stanford University, Stanford, CA USA; 9 Psychiatry, Yale University, New Haven, CT USA; 10 Biomedical Data Science, Stanford University, Stanford, CA USA; 11 Radiology, Stanford University, Stanford, CA USA; 12 Epidemiology & Population Health, Stanford University, Stanford, CA USA; 13 Chan Zuckerberg Biohub, San Francisco, CA 94158 USA

**Keywords:** Plasma, Biomarkers, Alzheimer’s disease, Phospho-tau

## Abstract

**Background:**

The recent promise of disease-modifying therapies for Alzheimer’s disease (AD) has reinforced the need for accurate biomarkers for early disease detection, diagnosis and treatment monitoring. Advances in the development of novel blood-based biomarkers for AD have revealed that plasma levels of tau phosphorylated at various residues are specific and sensitive to AD dementia. However, the currently available tests have shortcomings in access, throughput, and scalability that limit widespread implementation.

**Methods:**

We evaluated the diagnostic and prognostic performance of a high-throughput and fully-automated Lumipulse plasma p-tau181 assay for the detection of AD. Plasma from older clinically unimpaired individuals (CU, *n* = 463) and patients with mild cognitive impairment (MCI, *n* = 107) or AD dementia (*n* = 78) were obtained from the longitudinal Stanford University Alzheimer’s Disease Research Center (ADRC) and the Stanford Aging and Memory Study (SAMS) cohorts. We evaluated the discriminative accuracy of plasma p-tau181 for clinical AD diagnosis, association with amyloid β peptides and p-tau181 concentrations in CSF, association with amyloid positron emission tomography (PET), and ability to predict longitudinal cognitive and functional change.

**Results:**

The assay showed robust performance in differentiating AD from control participants (AUC 0.959, CI: 0.912 to 0.990), and was strongly associated with CSF p-tau181, CSF Aβ42/Aβ40 ratio, and amyloid-PET global SUVRs. Associations between plasma p-tau181 with CSF biomarkers were significant when examined separately in Aβ+ and Aβ− groups. Plasma p-tau181 significantly increased over time in CU and AD diagnostic groups. After controlling for clinical diagnosis, age, sex, and education, baseline plasma p-tau181 predicted change in MoCA overall and change in CDR Sum of Boxes in the AD group over follow-up of up to 5 years.

**Conclusions:**

This fully-automated and available blood-based biomarker assay therefore may be useful for early detection, diagnosis, prognosis, and treatment monitoring of AD.

**Supplementary Information:**

The online version contains supplementary material available at 10.1186/s13195-022-01116-2.

## Background

Alzheimer’s disease (AD) is the leading cause of dementia worldwide and global dementia prevalence is forecasted to nearly triple by 2050 [1]. Pathologically, AD is characterized by the accumulation of β-amyloid-containing plaques and the presence of intraneuronal neurofibrillary tangles comprised of hyperphosphorylated tau protein [2]. These pathological changes occur decades prior to symptom onset and clinical diagnosis [3]. Accordingly, accurate and early AD diagnosis is important for therapeutic intervention and outcomes. As such, there is a critical need for biomarkers that detect early signs of AD during the biochemical phase of the disease, such as among clinically unimpaired (CU) individuals with initial amyloid and tau accumulation (e.g., preclinical AD) [4].

Although cerebrospinal fluid (CSF) biomarkers and PET imaging can be used to detect early AD pathology, their invasiveness and cost preclude widescale application. These limitations have recently been overcome with development of novel AD-specific blood biomarkers, where plasma levels of tau phosphorylated at various residues have been demonstrated to be specific and sensitive biomarkers of AD in CU individuals and individuals with mild cognitive impairment and AD dementia [5–14]. However, currently employed plasma p-tau assays often use low throughput and plate-based approaches that limit scalability. Further, some of these prototype assays rely on proprietary reagents or are not yet commercially available, further limiting their widespread implementation [12]. As a fully-automated assay, the Lumipulse plasma p-tau181 test has the potential to be a high-throughput, fully-automated and available plasma p-tau test for AD.

In this cross-sectional and longitudinal study, we evaluate the diagnostic and prognostic accuracy of the Lumipulse plasma p-tau181 assay in older adults spanning the continuum from CU to MCI to AD. Outcome measures included diagnostic accuracy in distinguishing AD from CU, associations with amyloid and tau burden as measured in CSF and PET, and the ability to predict longitudinal cognitive and functional decline.

## Methods

### Study participants

Study protocols were approved by the Institutional Review Board of Stanford University. In accordance with the Declaration of Helsinki, written informed consent was obtained from each study participant or their legally authorized representative. Plasma from older clinically unimpaired individuals (CU, *n* = 463) and patients with mild cognitive impairment (MCI, *n* = 107) or AD dementia (*n* = 78) were obtained from the longitudinal Stanford University Iqbal Farrukh and Asad Jamal Alzheimer’s Disease Research Center (ADRC) and the Stanford Aging and Memory Study (SAMS) cohorts. Participants from the longitudinal Stanford ADRC cohort included 271 CU, 107 patients with a clinical diagnosis of MCI and 78 patients with a clinical diagnosis of AD dementia. Stanford ADRC participants undergo neurological examination, neuropsychological testing, and neuroimaging, and provide blood and CSF samples. Volunteers are asked to return each year for follow-up testing. An additional 192 CU participants were included from the SAMS cohort. SAMS is an ongoing prospective study of memory in CU older adults that seeks to understand how individual differences in memory relate to brain structure, brain function, and molecular and genetic risk for AD, with a key goal of identifying predictors of memory decline and dementia [15, 16]. SAMS eligibility included normal or corrected-to-normal vision/hearing, righthandedness, native English speaking, no history of neurologic or psychiatric disease, a Clinical Dementia Rating (CDR) global score of zero, performance within the normal range on a standardized neuropsychological test battery. SAMS participants undergo lumbar puncture to collect CSF and complete two MRI scanning sessions on a 3T and 7T scanner. For both ADRC and SAMS cohorts, diagnosis was determined at a clinical consensus meeting by a panel of neurologists and neuropsychologists.

### Assessment of global cognitive function

For Stanford ADRC participants, global cognitive function was assessed using the total scores from the Montreal Cognitive Assessment (MoCA) test [17], while Clinical Dementia Rating (CDR®) scale sum of boxes score was used to stage dementia severity based on cognition and function [18]. SAMS participants were not included in analyses of global cognitive function, as MoCA and CDR sum of boxes scores were not available in this cohort.

### CSF collection and analysis

CSF from both studies was collected by lumbar puncture and stored in externally threaded Thermo Scientific™ Nalgene™ General Long-Term Storage Cryogenic Tubes. CSF Aβ42, Aβ40, p-tau181, and total tau were measured by the Stanford ADRC Biomarker Core using the Lumipulse *G* assays (Fujirebio) on the LUMIPULSE *G*1200 instrument, as previously described [19]. The CSF amyloid β ratio cutpoint was determined using the Youden method to maximize sensitivity and specificity in discriminating clinically-defined AD from CU in the Stanford ADRC population. A CSF Aβ42/Aβ40 ratio < 0.091 was found to indicate amyloid pathology in this cohort. Following published guidelines set by the Consensus of the Task Force on Biological Markers in Psychiatry of the World Federation of Societies of Biological Psychiatry, CSF samples were subjected to a maximum of two freeze-thaw cycles [20].

### Lumipulse plasma p-tau181 analysis

Plasma p-tau181 levels were measured on a modified version of the Lumipulse *G* CSF p-Tau181 assay for CSF (Cat. # 231654, Fujirebio Diagnostics, US, Malvern, PA) using the LUMIPULSE *G*1200 instrument as previously described [21]. The Lumipulse *G* plasma p-tau181 assay antibody combination is based on the well-established and widely-used INNOTEST assay targeting tau epitopes proximal to Thr181, including antibody AT270 for capture, with HT7 and BT2 used for detection [22–24]. Briefly, plasma samples were thawed on wet ice, centrifuged for 5 min at 4°C at 500 × g before being loaded on the fully automated LUMIPULSE G1200 instrument. To minimize potential for non-specific binding, plasma samples were mixed with a heterophilic blocking reagent (200 μg/ml, Scantibodies Inc., Santee, CA) prior to measurement, as previously described [6, 10]. Individual-level variability was assessed on 6 independent plasma aliquots using a different lot of reagents one year later and showed high test-retest reliability (Pearson’s *r* = 0.98). 100% of plasma samples from the current study fell within the quantifiable range (range 0.46–11.47 pg/ml). Samples were tested by experimenters blind to diagnostic information.

### Amyloid PET imaging

Amyloid PET scanning with ^18^F-Florbetaben was completed at the Richard M. Lucas Center for Imaging at Stanford University, using a simultaneous time-of-flight (TOF)–enabled PET/MRI scanner (Signa 3 T, GE Healthcare). Emission data was collected between 90 and 110 min following an 8.1mCi (mean) injection of ^18^F-Florbetaben. PET data were reconstructed into 5-min frames using standard methods, with zero-TE (ZTE) MR imaging for MR attenuation correction (MRAC) and 5-min frame data were realigned and summed. FreeSurfer v7 regions of interests (ROIs) from the Desikan aparc+aseg atlas were defined on each participant’s MRI and used to extract intensity values from the co-registered summed PET data. Standardized uptake value ratios (SUVRs) were created for a global ROI (an average across frontal, parietal, lateral temporal, and cingulate) using a whole cerebellum reference region [25]. The mean delay between the amyloid PET scan and plasma draw was 9.4 weeks (range 0–29.6 weeks). Amyloid-positivity was determined by qualitative reads, using a consensus rating of three trained readers blind to clinical information.

### Statistical analysis

Given the skewness in the distributions of CSF biomarkers, concentrations were log_10_-transformed to meet the assumptions of Gaussian distribution for the relevant statistical analyses. Differences between clinical diagnostic groups were first examined. Continuous variables were compared using one-way ANOVA. Categorical variables were assessed through chi-square tests. To determine whether plasma p-tau181 differed between clinical diagnostic groups, log_10_-transformed plasma p-tau181 levels were analyzed using ANCOVA with diagnosis as the independent variable of interest, while age and gender were included as covariates. Receiver operating characteristic (ROC) curve analysis was performed to summarize the ability of plasma p-tau181 in differentiating diagnostic subgroups. Tukey corrected post hoc pairwise comparisons were applied to examine differences between diagnostic groups. The nonparametric Mann-Whitney *U* test or Kruskal-Wallis test followed by Dunn’s corrected post hoc comparisons (non-parametric) were applied to examine differences between diagnostic groups as a sensitivity analysis.

Plasma p-tau181 associations with CSF and PET biomarkers at baseline were evaluated using linear regression models and controlling for age and sex. Linear regression models were also used to examine the effects of baseline plasma p-tau181, clinical diagnosis group, and their interaction on baseline global cognition after controlling for age, sex, and education. This analysis was repeated for CDR-SB.

Linear mixed models were used to examine (1) longitudinal change in plasma p-tau181, (2) whether baseline plasma p-tau181 predicted change in global cognition (MoCA), and (3) whether baseline plasma p-tau181 predicted change in function (CDR-SB). For the assessment of longitudinal change in plasma p-tau181, we conducted a linear mixed model with fixed effects of clinical diagnosis (CU, MCI, AD) at the time of the blood draw, time from initial blood draw, and their interaction, as well as a random intercept to account for within-subject correlations. In this model, contrasts were examined to determine whether change in plasma p-tau181 was (1) significantly different than zero within each clinical diagnostic group and (2) whether change over time differed across diagnostic groups. This regression analysis was repeated with additional adjustment of age, sex, and their interactions with time since the initial blood draw. Of the 219 participants with more than one plasma measure (range = 2–5), the mean follow-up time between their first and last blood draw was 2.05 years (SD = 1.03) for CU, 1.99 years (SD = 0.90) for MCI, and 1.86 years (SD = 0.71) for AD.

Specifically, we fitted the following two mixed effects regression models:

$${Y}_{ij}={\beta}_1+{\beta}_2^{\prime }{X}_{i,\mathit{\operatorname{diag}}}+\left({\beta}_3+{\beta}_4^{\prime }{X}_{i,\mathit{\operatorname{diag}}}\right)\times {t}_{ij}+{b}_i+{e}_{ij}$$

and

$${Y}_{ij}={\beta}_1+{\beta}_2^{\prime }{X}_{i,\mathit{\operatorname{diag}}}+{\beta}_3 Ag{e}_i+{\beta}_4 Se{x}_i+\left({\beta}_5+{\beta}_6^{\prime }{X}_{i,\mathit{\operatorname{diag}}}+{\beta}_7 Ag{e}_i+{\beta}_8 Se{x}_i\right)\times {t}_{ij}+{b}_i+{e}_{ij}$$

where

*Y*_*ij*_ is the Plasma p-tau181 level at the *j*th visit for the *i*th participant,

*t*_*ij*_ is the time of the *j*th plasma measurement relative to the first plasma measurement (in years) for the *i*th participant,

*X*_*i*, *diag*_ is the baseline diagnosis group for the *i*th participant (CU, MCI, and AD),

*b*_*i*_ is the random intercept for the *i*th participant, and

*e*_*ij*_ is the mean zero random error at the *j*th visit of the *i*th participant.

To examine the association between baseline plasma p-tau181 with change in global cognition, we first used mixed regression models including a random intercept and time from initial MoCA, clinical diagnosis (CU, MCI, and AD) at the time of blood draw, age at time of blood draw, sex, education, and their interactions with time as predictors. In this analysis, the interaction between baseline plasma p-tau181 and time indicated whether plasma p-tau181 levels were associated with change in cognition after controlling for demographics and clinical diagnosis. To determine the associations between plasma p-tau181 and change in cognition within each clinical diagnosis as well as whether the association differs between diagnoses, we conducted a second mixed effects regression analysis that additionally included a three-way interaction term between clinical diagnosis, baseline plasma p-tau181, and time. Specifically, we fitted the following two regression models:

Model 1

$${Y}_{ij}={\beta}_1+{\beta}_2 Ag{e}_i+{\beta}_3 Se{x}_i+{\beta}_4 Educatio{n}_i+{\beta}_5^{\prime }{X}_{i,\mathit{\operatorname{diag}}}+{\beta}_6{X}_{i,P-\tau 181}+\left({\beta}_7+{\beta}_8 Ag{e}_i+{\beta}_9 Se{x}_i+{\beta}_{10} Educatio{n}_i+{\beta}_{11}^{\prime }{X}_{i,\mathit{\operatorname{diag}}}+{\beta}_{12}{X}_{i,P-\tau 181}\right)\times {t}_{ij}+{b}_i+{e}_{ij}$$

and

Model 2

$${Y}_{ij}={\beta}_1+{\beta}_2 Ag{e}_i+{\beta}_3 Se{x}_i+{\beta}_4 Educatio{n}_i+{\beta}_5^{\prime}\log {X}_{i,\mathit{\operatorname{diag}}}+{\beta}_6{X}_{i,P-\tau 181}+\left({\beta}_7+{\beta}_8 Ag{e}_i+{\beta}_9 Se{x}_i+{\beta}_{10} Educatio{n}_i+{\beta}_{11}^{\prime }{X}_{i,\mathit{\operatorname{diag}}}+{\beta}_{12}{X}_{i,P-\tau 181}+{\beta}_{13}{X}_{i,\mathit{\operatorname{diag}}}\times \log \left({X}_{i,P-\tau 181}\right)\right)\times {t}_{ij}+{b}_i+{e}_{ij}$$

where

*Y*_*ij*_ is the MoCA total scores at the *j*th visit of the *i*th participant,

Age_i_ is the centered age for the *i*th participant at the first blood draw,

Sex_i_: is the sex (male, female) of the *i*th participant,

Education_i_ is the centered education in years for the *i*th participant,

*X*_*i*, *diag*_ is the clinical diagnosis closest to first blood draw (CU, MCI, AD) for the *i*th participant,

*X*_*i*, *P* − *τ*181_ is the plasma p-tau181 level for the *i*th participant at the first blood draw,

*t*_*ij*_ is the time in years of the *j*th visit since the first MoCA assessment within 1 year of the first blood draw for the *i*th participant,

*b*_*i*_ is the random intercept for the *i*th participant, and

*e*_*ij*_: the mean zero random error

These models were repeated with CDR sum of boxes as the outcome (replacing MoCA total score) to examine the relationship with plasma p-tau181. Statistical tests were two-sided at a significance level of α = 0.05. Tests were performed using GraphPad Prism software and R.

## Results

### Participant characteristics

The study cohort included CU (*n* = 463), MCI (*n* = 107), and AD (*n* = 78) participants. Demographic characteristics of study participants are presented in Table 1. Participants in the MCI group (73.0 ± 7.5 years {mean ± SD}) were older than those in the CU group (69.9 ± 7.8 years) (*p* < 0.001) and the AD group (70.1 ± 10.4 years) (*p* < 0.05). The participant groups differed in gender distribution (*p* < 0.01), with the CU group overrepresented with women while the MCI group included more men.

**Table 1:** Participant characteristics by clinical diagnosis

	CU (***n*** = 463)	MCI (***n*** = 107)	AD (***n*** = 78)	***p*** value
**Age, years**	69.9 ± 7.8	73.0 ± 7.5^a^	70.1 ± 10.4^b^	0.0014
**Sex, female**	278 (60.0)	45 (42.0)	41 (52.6)	0.0026
**Plasma p-tau181, pg/ml**	1.68 ± 0.71	2.08 ± 0.74^a^	3.02 ± 1.16^a,c^	0.001

*Abbreviations*: *AD* Alzheimer’s disease, *CU* clinically unimpaired, *MCI* mild cognitive impairment. Continuous variables expressed as mean ± SD while categorical values are expressed as *n* (%)

^a^, *** vs CU; ^b^, * vs MCI; ^c^, *** vs. MCI. Data analyzed using one-way ANOVA with Tukey’s post hoc tests for multiple comparisons (age), chi-square (sex), or by ANCOVA with age and sex included as covariates (plasma p-tau181).* *p* < 0.05, *** *p* < 0.001

### Lumipulse plasma p-tau181 concentration distinguishes AD from CU

**Fig. 1:**
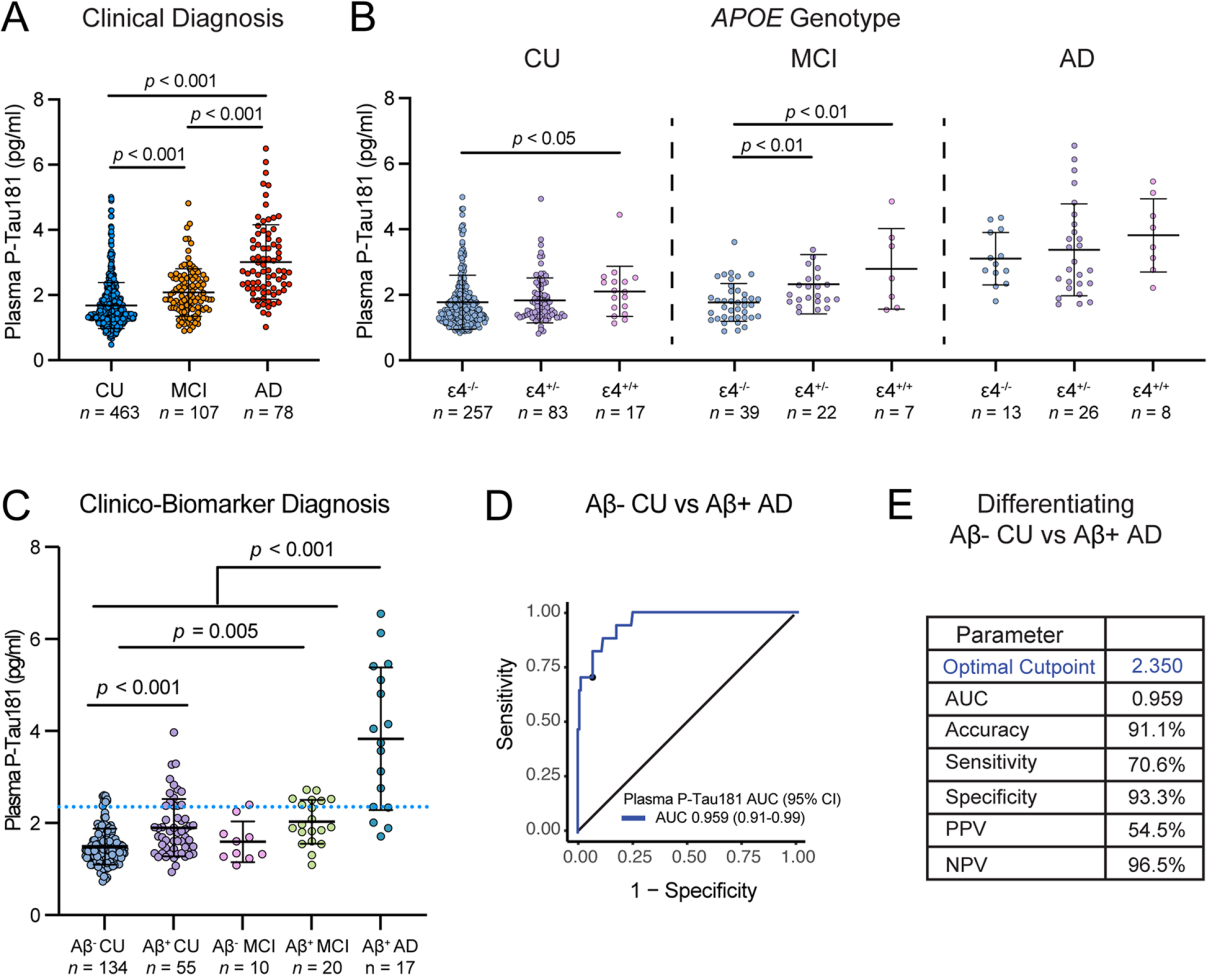
Plasma p-tau181 distinguishes Aβ− CU from Aβ+ AD. **A** Plasma p-tau181 was highest in AD (*n* = 78) followed by MCI (*n* = 107) and CU (*n* = 463) groups. **B** Plasma p-tau181 was higher in groups with increasing copy number of the *APOE* ε4 allele in CU and MCI. **C** Classification of participants by amyloid-positivity revealed plasma p-tau181 was elevated in Aβ+ CU and Aβ+ MCI as well as in Aβ+ AD. **D** ROC curve analysis of p-tau181 in distinguishing AD cases from Aβ− CU controls. **E** The Youden method was used to determine optimal cutpoint maximizing sensitivity and specificity for distinguishing Aβ+ AD cases from Aβ− CU. Log_10_-transformed plasma p-tau 181 data analyzed using ANCOVA with age and sex included as cofactors. Tukey’s post hoc test was used for pairwise comparisons (**A**–**C**). Data presented as mean ± SD

Analysis of Lumipulse plasma p-tau181 across diagnostic groups revealed that concentrations were highest in AD participants followed by MCI and then CU (*p* all < 0.001) (Fig. 1A). The subset of participants with *APOE* genotype available (participant characteristics provided in Table S1) showed that *APOE* ε4 allele carriage was associated with higher plasma p-tau181 levels within both the CU and MCI groups (Fig. 1B). In both diagnostic groups CU and MCI, higher plasma p-tau181 was observed in the group of participants with two copies of the *APOE* ε4 allele when compared to the non-ε4 carrier group (*p* < 0.05 and *p* < 0.01, respectively). In MCI, a single copy of the *APOE* ε4 allele was sufficient to produce a group-level increase in plasma p-tau181 levels (*p* < 0.01). Importantly, the association between plasma p-tau181 and *APOE* ε4 was lost when amyloid (CSF Aβ42/A40) was included in the regression model, suggesting that this association may be driven by the range of amyloid pathology within each diagnostic group. No significant differences were observed in the AD diagnostic group, perhaps owing to a possible ceiling effect in plasma p-tau181 levels in that group.

We next stratified the study population by amyloid status (Aβ+ or Aβ− based on CSF Aβ peptide data—see the “Methods” section) to focus on 134 Aβ− CU, 55 Aβ+ CU, 10 Aβ− MCI, 20 Aβ+ MCI, and 17 Aβ+ AD participants (participant characteristics provided in Table S2). Accordingly, the Aβ+ AD group showed significantly higher plasma p-tau181 levels compared to all other groups (all *p* < 0.001) (Fig. 1C). Furthermore, plasma p-tau181 was higher in Aβ+ CU compared to Aβ− CU participants (*p* < 0.001). Similarly, plasma p-tau181 was greater in Aβ+ MCI compared to Aβ− CU (*p* = 0.005) and did not differ from Aβ+ CU (*p* > 0.05). ROC analysis revealed plasma p-tau181 distinguished Aβ+ AD from Aβ− CU group with an AUC of 0.959 (CI: 0.912 to 0.990) (Fig. 1D), corresponding to an optimal cut-point (maximizing sensitivity and specificity) of 2.350 pg/ml (Fig. 1E). ROC analyses also showed plasma p-tau181 differentiating Aβ+ AD from Aβ+ CU with an AUC = 0.886 (CI: 0.784 to 0.966). At the same time, plasma p-tau181 differentiated Aβ+ MCI from Aβ− CU with an AUC = 0.771 (CI: 0.668 to 0.872) and differentiated the Aβ+ CU group from the Aβ− CU group with an AUC = 0.669 (CI: 0.582 to 0.749). Notably, there was poor discrimination between MCI and Aβ+ CU (AUC = 0.492; CI: 0.349 to 0.623). These ROC analyses suggest the plasma p-tau181 level more closely reflects the Aβ+ vs Aβ− distinction, rather than the clinical distinction. Applying the cut point for plasma p-tau181-positivity, we found 21.5% of the overall study population was plasma p-tau181-positive (62.1% of whom were also Aβ+), 24.1% of the MCI group (85.7% Aβ+) and 76.2% of the AD group (81.3% Aβ+) were positive for plasma p-tau181. Furthermore, 11.8% of the CU group (53.8% Aβ+) were plasma p-tau181-positive. Taken together, these results highlight the diagnostic accuracy of the Lumipulse plasma p-tau181 assay in distinguishing clinical AD as well as in detecting AD-related change in non-symptomatic (CU) and mildly symptomatic (MCI) groups.

### Relationship between concentration of plasma p-tau181 with CSF amyloid β peptides and tau

**Fig. 2:**
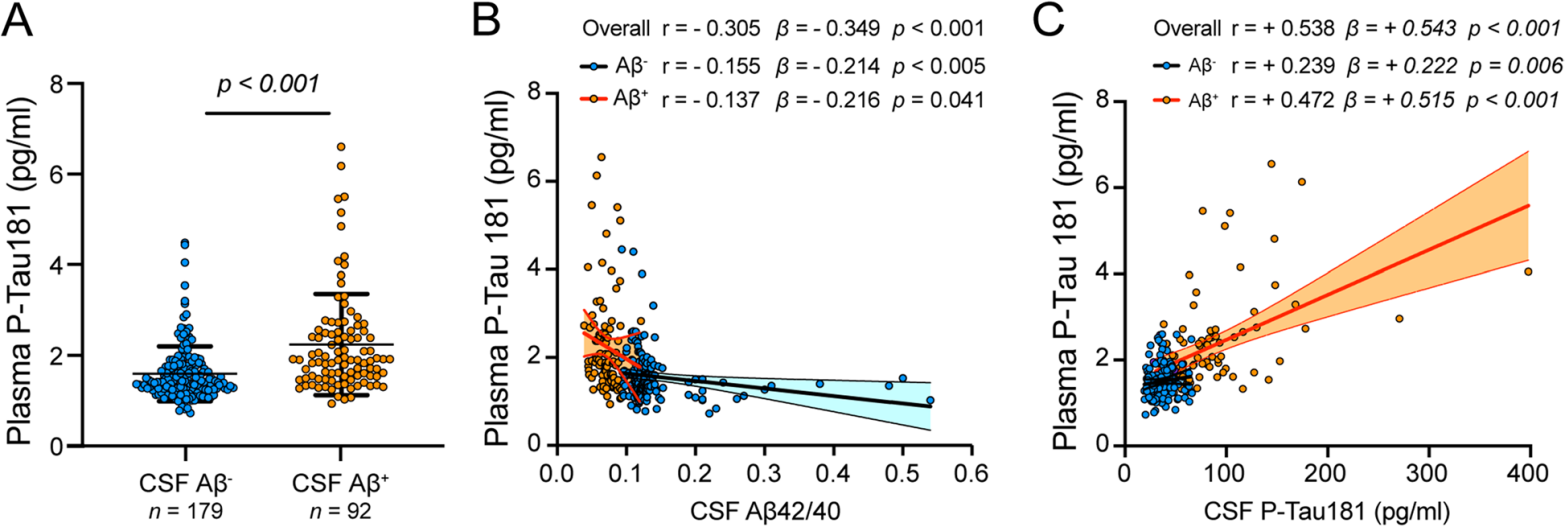
Plasma p-tau181 associates with amyloid and tau CSF biomarkers. **A** Plasma p-tau181 was higher in CSF Aβ+ (*n* = 92) compared to Aβ− participants (*n* =179). **B** Plasma p-tau181 was positively associated with CSF Aβ42/Aβ40 ratio overall and after stratifying participants according to CSF Aβ+ (orange circles), as well as in Aβ− participants (blue circles). **C** Plasma p-tau181 was significantly associated with CSF p-tau181 overall and again in both Aβ+ and Aβ− participants. Raw data are presented as mean ± SD (**A**) or plotted with the 95% confidence band of the best-fit line with Pearson correlation coefficient r, and β estimates and *p*-value from linear regression models with age and sex as covariates (**B** and **C**). *n* = 92 (CSF Aβ+) and *n* = 179 (CSF Aβ−)

In this same subset of participants with CSF amyloid and tau data described above, we found that CSF Aβ+ participants had significantly higher plasma p-tau181 compared to Aβ− participants when controlling for age and sex (*p* < 0.001) (Fig. 2A). When examining continuous effects of amyloid, plasma p-tau181 was significantly associated with CSF Aβ_42_/Aβ_40_ (β = −0.349, *p* < 0.001) and this association remained significant when examined in Aβ+ and Aβ− groups separately (β = −0.216, *p* < 0.05 and β = −0.214, *p* < 0.01, respectively) (Fig. 2B). Further analysis revealed plasma p-tau181 also positively associated with CSF p-tau181 (β = +0.543, *p* < 0.001) (Figure 2C). This association was significant when examined in Aβ+ and Aβ− groups separately (β = +0.515, *p* < 0.001 and β = +0.222, *p* < 0.01, respectively).

### Relationship between plasma p-tau181 concentration and amyloid PET

**Fig. 3:**
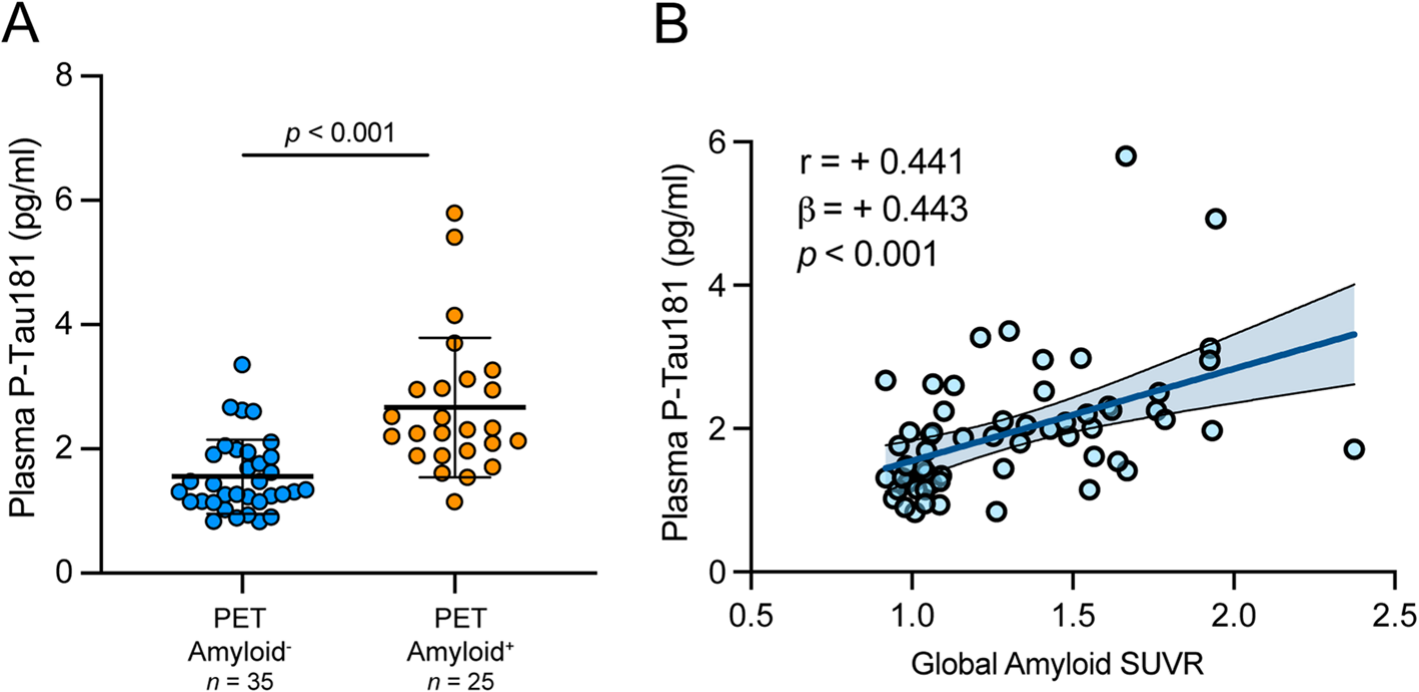
Plasma p-tau181 associates with amyloid PET. Amyloid PET neuroimaging with Florbetaben revealed that plasma p-tau181 was significantly higher in amyloid PET+ (*n* = 25) compared to amyloid PET− (*n* = 35) participants (**A**) and positively associated with global amyloid PET (**B**). Raw data are presented as mean ± SD (**A**) or plotted with the 95% confidence band of the best-fit line with Pearson correlation coefficient r, and β estimates and *p*-value from linear regression models with age and sex as covariates (**B**)

A subset of 37 CU, 16 MCI and 7 AD participants had undergone amyloid PET imaging with ^18^F-Florbetaben. PET Aβ+ participants showed significantly higher plasma p-tau181 compared to PET Aβ− participants after controlling for age and sex (*p* < 0.001) (Fig. 3A). Examination of continuous relationships further showed that plasma p-tau181 was positively associated with global amyloid SUVR after controlling for age and sex (β = +0.443, *p* < 0.001) (Fig. 3B).

### Longitudinal change in Lumipulse plasma p-tau181 concentration

**Fig. 4:**
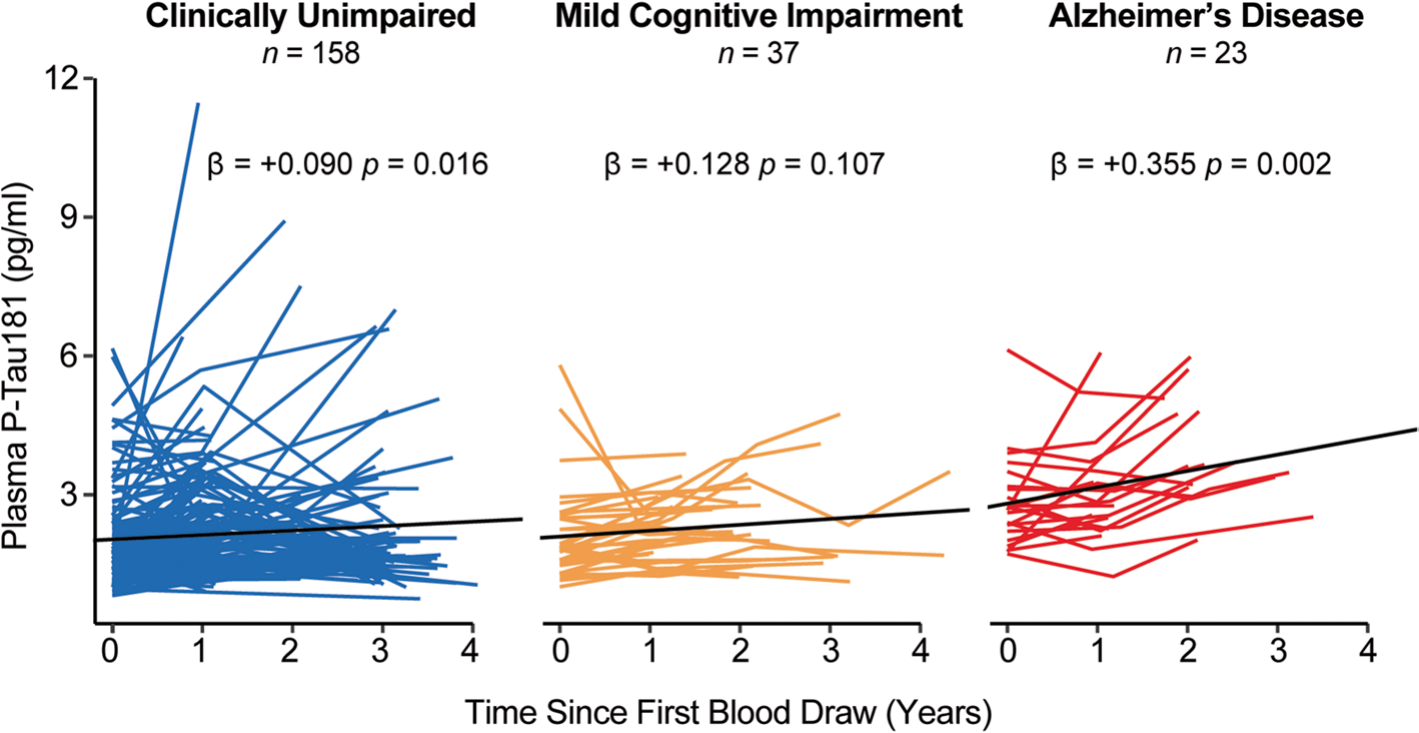
Plasma p-tau181 increases with time in CU and AD diagnostic groups. Change in plasma p-tau181 over time was significantly different than zero in CU and AD diagnostic groups. Shown are the *p*-values and the unstandardized regression co-efficient (β)

One hundred fifty-eight CU (mean follow-up: 2.05 years ± 1.03, range: 0.66 to 4.33 years), 37 MCI (1.99 years ± 0.90, range: 0.86 to 4.33 years), and 23 AD (1.86 years ± 0.71, range: 0.94 to 3.39 years) had 2 to 5 plasma follow up measurements. Plasma p-tau181 significantly increased over time for CU (β = +0.090 pg/ml/year, *p* = 0.016) and AD (β = +0.355 pg/ml/year, *p* = 0.002) diagnostic groups and increased significantly more in AD than CU individuals (Fig. 4 and Table S3). The increase in plasma p-tau181 in MCI did not differ from CU or AD. The effects were similar when additionally accounting for age and sex (Table S3).

### Plasma p-tau181 concentration predicts cross-sectional cognition and function

**Fig. 5:**
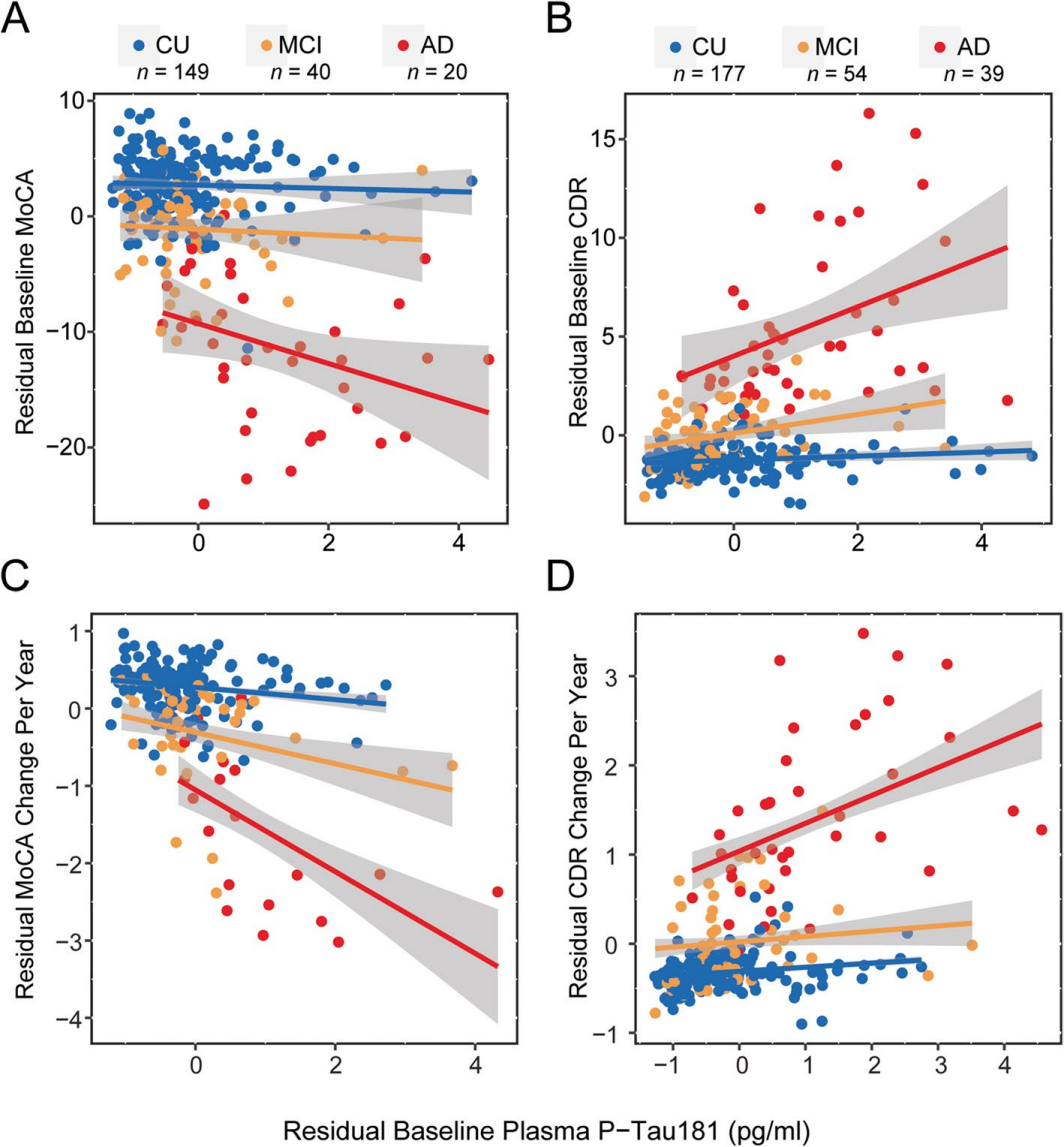
Plasma p-tau181 associates with cognitive and functional measures of AD. **A** Higher baseline plasma p-tau181 was associated with worse baseline cognition measured by the MoCA for the AD group only and this association was significantly stronger for AD compared to CU and MCI groups. **B** Higher baseline plasma p-tau181 was associated with worse baseline functioning measured by the CDR for MCI and AD groups, and this association was significantly stronger for AD compared to CU and MCI groups. **C** Higher baseline plasma p-tau181 was associated with greater cognitive decline overall and this association was stronger for MCI than CU. **D** Higher baseline plasma p-tau181 was associated with greater functional decline in the AD group. All depicted residual values were controlled for age, sex, and education; change in MoCA and CDR per year was calculated for visualization purposes only

At baseline, higher plasma p-tau181 concentration was associated with worse global cognition, assessed by MoCA, in AD (β (SE) = −1.661 (0.458), *p* < 0.001) after adjusting for age, sex, and education (Fig. 5A; Table S4). Baseline plasma p-tau181 was not significantly associated with global cognition for CU or MCI groups. The association between baseline plasma p-tau181 and baseline global cognition was significantly stronger in AD compared to CU and MCI groups, but did not differ between MCI and CU groups (Table S4). Thus, the association between plasma p-tau181 and cognition is present and most strongly observed in AD individuals.

Higher plasma p-tau181 concentration at baseline was also associated with worse CDR-SB scores at baseline for MCI (β (SE) = +0.449 (0.225), *p* = 0.047) and AD (β (SE) = +1.336 (0.196), *p* < 0.001) groups after accounting for age, sex, and education (Fig. 5B; Table S4). This association was not significant for CU. The association between baseline plasma p-tau181 and baseline functioning was significantly stronger in AD compared to CU and MCI, and marginally significant for MCI compared to CU. Thus, plasma p-tau181 concentration is related to functional status in a stepwise manner with the strongest associations in AD then MCI and CU.

### Plasma p-tau181 concentration predicts prospective decline in cognition and function

Longitudinal global cognitive and functional decline was assessed in participants with 2 or more MoCA or CDR measurements, respectively. 149 CU (mean cognitive follow-up: 2.33 years ± 1.36, range: 0.78 to 5.31 years), 39 MCI (2.08 years ± 1.03, range: 0.89 to 5.23 years), and 20 AD (1.90 years ± 0.91, range: 0.94 to 4.19 years) had 2 to 5 MoCA assessments. Higher baseline plasma p-tau181 concentration predicted decline in global cognition, as measured by MoCA, over time (β (SE) = −0.173 change in MoCA/year (0.087), *p* = 0.048) and these effects were independent of clinical diagnosis and demographics. Within the diagnostic groups, a higher baseline plasma p-tau181 concentration significantly predicted decline in global cognition in MCI (β (SE) = −0.748 change in MoCA score/year (0.178), *p* < 0.001) and the association between plasma p-tau181 levels and longitudinal decline in cognition was significantly stronger in MCI compared to CU (Fig. 5C; Table S5). Plasma p-tau181 concentration did not significantly predict cognitive decline in CU or AD. There was no significant difference between AD and CU or AD and MCI. Thus, plasma p-tau181 levels significantly predicted cognitive decline and this effect was most pronounced amongst MCI.

One hundred seventy-seven CU (mean follow-up: 3.42 years ± 1.36, range: 0.99 to 5.19 years), 54 MCI (2.85 years ± 1.25, range: 0.88 to 6.13 years), and 39 AD (2.65 years ± 1.33, range: 0.54 to 6.09 years) had 2 to 7 CDR assessments. Higher baseline plasma p-tau181 concentration did not significantly predict decline in function over time overall (β (SE) = 0.037 (0.021), *p* = 0.086), and these effects were independent of clinical diagnosis and demographics. However, within the diagnostic groups, higher baseline plasma p-tau181 significantly predicted decline in CDR-SB in AD (β (SE) = +0.300 change in CDR-SB score/year (0.060), *p* < 0.001) and this association was stronger in AD compared to CU and MCI groups (Fig. 5D; Table S5). Plasma p-tau181 did not significantly predict functional decline in CU or MCI, and this association did not significantly differ between CU and MCI groups. Thus, plasma p-tau181 levels predicted functional decline amongst individuals with AD.

## Discussion

The current study examined the diagnostic and prognostic ability of the Lumipulse plasma p-tau181 assay run on a fully-automated platform in participants spanning the AD continuum from the Stanford Alzheimer’s Disease Research Center and Stanford Aging and Memory Study. Consistent with previous work, we showed that cross-sectional plasma p-tau181 was increased in MCI and AD participant groups compared to the CU group. Participant stratification by CSF amyloid revealed that this observation was mainly driven by the proportion of Aβ+ in each group, since it was found that the CU Aβ+ did not differ from MCI. Thus, plasma p-tau181 was elevated in Aβ+ subgroups and was positively associated with continuous measures of amyloid PET and CSF Aβ42/40 ratio. Plasma p-tau181 was also associated with continuous CSF p-tau181, even within the CU group. Plasma p-tau181 measured longitudinally increased significantly in the CU and AD groups. Finally, baseline plasma p-tau181 predicted prospective cognitive decline overall and functional decline in the AD group.

Our findings are also consistent with previous studies showing a gradual increase in plasma p-tau181 concentrations with progression along the AD continuum [5, 6, 10, 26]. In an analysis similar to that of the current study, the University of Gothenburg’s in-house Simoa assay for plasma p-tau181 discriminated Aβ+ AD (*n* = 137) from Aβ− CU (*n*=268) in Alzheimer’s Disease Neuroimaging Initiative (ADNI) participants, with an AUC = 0.853 [7]. A related study by Karikari and colleagues in the BioFINDER-2 cohort showed the assay differentiated AD from Aβ− cognitively unimpaired controls with an AUC of 0.902 [26]. In the current study, ROC curve analysis revealed the Lumipulse plasma p-tau181 assay distinguished AD dementia from Aβ− CU with an AUC of 0.959. Notably, the Lumipulse plasma p-tau181 assay significantly predicted CSF p-tau181 and amyloid, not only in Aβ+ groups, but also in Aβ− groups. This suggests that Lumipulse plasma p-tau181 may be used to estimate CSF amyloid and tau levels even within lower concentration ranges. Beyond CSF biomarkers, plasma p-tau181 also significantly predicted global amyloid PET SUVR.

Time course analysis of plasma p-tau181 in the ADNI cohort showed that it becomes abnormal 6.5 and 5.7 years after the detection of abnormal CSF and PET amyloid, respectively [27]. Yet, in familial AD plasma p-tau181 begins to increase 16 years prior to estimated symptom onset [28]. Thus, plasma p-tau181 may be a beneficial diagnostic and screening tool for AD within the wide window after amyloid has become abnormal but before clinical symptoms. One could anticipate the benefits whereby plasma p-tau181-positive individuals might be referred for additional clinical evaluation — either through biomarker and/or neuropsychological examination — and to initiate early treatment.

Assessing plasma p-tau181 rate of change over an approximately 24-month period, we detected an overall positive change in the CU and AD groups, with the increase significantly greater in the AD group compared to the CU group. Longitudinal increases in plasma p-tau181 have been demonstrated in Alzheimer’s ADNI participants, where Moscoso and colleagues showed dynamic changes in plasma p-tau181 before PET and CSF amyloid reach abnormal levels, and accelerating with increasing amyloid-β pathology [27]. Our study also demonstrated that elevated baseline Lumipulse plasma p-tau181 predicted prospective decline in cognitive measures of AD. This is consistent with results showing that baseline levels of plasma p-tau181 were associated with faster rates of memory decline over periods spanning 1 to 5 years [26, 29–31]. In this context, our results provide further support for the sensitivity of the plasma p-tau181 assay in detecting early and subtle cognitive changes in AD development. We add to previous findings by demonstrating that plasma p-tau181 is a predictor of functional decline as measured by change in CDR.

Beyond phosphorylation at Thr181, several other tau phosphorylation sites have been found to be sensitive to AD, including Thr217 [32, 33] and Thr231 [14]. Previous work has shown that plasma p-tau217 has higher discriminative accuracy for AD compared to p-tau181 [5, 34] and a higher dynamic range [35]. More recently, studies have shown plasma p-tau231 increases earlier compared to plasma p-tau181 [13, 14] and associates more strongly with tau and Aβ-PET in presymptomatic individuals [11]. However, head-to-head comparison indicated that the plasma p-tau217 assay provided higher diagnostic accuracy for the diagnosis of AD dementia [35]. While it is impossible to directly compare the performance of Lumipulse plasma p-tau181 to these assays, a recent framework to validate plasma tau biomarkers for AD diagnosis has been developed [36, 37] and proper head-to-head comparisons between the various assays are currently underway. It is possible that there are subtle differences across p-tau species detected by these analysis platforms [38], raising the exciting possibility that if performed in parallel, they may provide a more complete understanding of the pathological state and better predict disease trajectory.

While this study demonstrates the high diagnostic and prognostic value of the Lumipulse plasma p-tau181 assay for AD, this study has several limitations. First, our data have been obtained from a single site at Stanford University, and therefore multi-site comparisons will need to be performed to determine the site-to-site variability in the assay. For example, it is unlikely that our cut-points will perform well at other centers or while examining other cohorts, and it is instead more likely that center-specific cutoffs will be needed. Second, our study lacked other plasma p-tau isoforms. Notably, it would be important to investigate what additional information could be provided from plasma p-tau217 and p-tau231. Third, it will be important to assess associations between baseline plasma p-tau181 concentration and the rate of conversion to AD dementia. Finally, we did not examine race effects as our study incorporated a mostly homogeneous non-Hispanic White population. In this regard, however, implementation of plasma p-tau181 may help alleviate some of the barriers to the inclusion of more diverse populations. For example, despite recent advances in blood-based biomarkers, the recommended gold standard for AD diagnosis remains through amyloid and tau PET neuroimaging [39] and the associated cost and the required expertise for PET are often prohibitive [40]. Invasiveness of CSF collection is another barrier dissuading participants from AD research and the minimally-invasive nature of plasma sampling may further support participant recruitment [41, 42]. In these ways, plasma p-tau181 measurement may serve to improve recruitment efforts to diversify research in support of expanding equitable medicine.

To conclude, in a cohort of participants from the Stanford ADRC and Stanford Aging and Memory Study, we demonstrate that the Lumipulse plasma p-tau181 assay run on a fully-automated and high-throughput platform discriminated AD with a high degree of accuracy, associated with CSF and PET AD biomarkers, and predicted AD-related measures of prospective cognitive and functional decline. While several prototype assays have been developed to measure plasma p-tau in clinical and research settings, many are not yet commercially available — a constraint limiting widespread implementation [12]. Our results demonstrate that the Lumipulse plasma p-tau181 is a high-throughput, fully-automated, highly scalable and accessible assay that is potentially useful for widescale early detection, diagnosis, and therapeutic monitoring of AD.

## Supplementary Information

**Additional file 1: Table S1.** Characteristics of Participants Stratified by Diagnosis and *APOE* Genotype.

**Additional file 2: Table S2.** Characteristics of Participants Stratified by Diagnosis and AD CSF Biomarkers.

**Additional file 3: Table S3.** Betas (standard errors) and *p* values within and between clinical groups for models examining longitudinal change in plasma p-tau181.

**Additional file 4: Table S4.** Betas (standard errors) and *p* values for models examining plasma p-tau181 and cross-sectional cognition and function.

**Additional file 5: Table S5.** Betas (standard errors) and *p* values for models examining plasma p-tau181 and longitudinal change in global cognition and function.

## Data Availability

Stanford ADRC and SAMS de-identified data are available to qualified researchers upon approved request to the Stanford ADRC and SAMS.

## Abbreviations

AD: Alzheimer disease
ADNI: Alzheimer’s Disease Neuroimaging Initiative
ADRC: Alzheimer’s Disease Research Center
AUC: Area under the curve
CDR-SB: Clinical Dementia Rating - Sum of Boxes
CI: Confidence interval
CU: Clinically unimpaired
LME: Linear mixed effects
MCI: Mild cognitive impairment
MoCA: Montreal Cognitive Assessment
NPV: Negative predictive value
p-tau: Phosphorylated tau
PET: Positron emission tomography
PPV: Positive predictive value
ROC: Receiver operating characteristic
SAMS: Stanford Aging and Memory Study
SD: Standard deviation
SUVR: Standardized uptake value ratio

## References

[1] Collaborators GBDDF (2022). Estimation of the global prevalence of dementia in 2019 and forecasted prevalence in 2050: an analysis for the Global Burden of Disease Study 2019. Lancet Public Health.

[2] Knopman DS, Amieva H, Petersen RC, Chetelat G, Holtzman DM, Hyman BT, Nixon RA, Jones DT (2021). Alzheimer disease. Nat Rev Dis Primers.

[3] Dubois B, Hampel H, Feldman HH, Scheltens P, Aisen P, Andrieu S, Bakardjian H, Benali H, Bertram L, Blennow K, Broich K, Cavedo E, Crutch S, Dartigues JF, Duyckaerts C, Epelbaum S, Frisoni GB, Gauthier S, Genthon R, Gouw AA, Habert MO, Holtzman DM, Kivipelto M, Lista S, Molinuevo JL, O'Bryant SE, Rabinovici GD, Rowe C, Salloway S, Schneider LS, Sperling R, Teichmann M, Carrillo MC, Cummings J, Jack CR (2016). Preclinical Alzheimer's disease: Definition, natural history, and diagnostic criteria. Alzheimers Dementia.

[4] Sperling R, Mormino E, Johnson K (2014). The evolution of preclinical Alzheimer's disease: implications for prevention trials. Neuron.

[5] Thijssen EH, La Joie R, Wolf A, Strom A, Wang P, Iaccarino L, Bourakova V, Cobigo Y, Heuer H, Spina S, VandeVrede L, Chai X, Proctor NK, Airey DC, Shcherbinin S, Duggan Evans C, Sims JR, Zetterberg H, Blennow K, Karydas AM, Teunissen CE, Kramer JH, Grinberg LT, Seeley WW, Rosen H, Boeve BF, Miller BL, Rabinovici GD, Dage JL, Rojas JC, Boxer AL, Advancing R, Treatment for Frontotemporal Lobar Degeneration i (2020). Diagnostic value of plasma phosphorylated tau181 in Alzheimer's disease and frontotemporal lobar degeneration. Nat Med.

[6] Janelidze S, Mattsson N, Palmqvist S, Smith R, Beach TG, Serrano GE, Chai X, Proctor NK, Eichenlaub U, Zetterberg H, Blennow K, Reiman EM, Stomrud E, Dage JL, Hansson O (2020). Plasma P-tau181 in Alzheimer's disease: relationship to other biomarkers, differential diagnosis, neuropathology and longitudinal progression to Alzheimer's dementia. Nat Med.

[7] Karikari TK, Benedet AL, Ashton NJ, Lantero Rodriguez J, Snellman A, Suarez-Calvet M (2021). Diagnostic performance and prediction of clinical progression of plasma phospho-tau181 in the Alzheimer's Disease Neuroimaging Initiative. Mol Psychiatry.

[8] Lantero Rodriguez J, Karikari TK, Suarez-Calvet M, Troakes C, King A, Emersic A, Aarsland D, Hye A, Zetterberg H, Blennow K, Ashton NJ (2020). Plasma p-tau181 accurately predicts Alzheimer's disease pathology at least 8 years prior to post-mortem and improves the clinical characterisation of cognitive decline. Acta Neuropathol.

[9] Suarez-Calvet M, Karikari TK, Ashton NJ, Lantero Rodriguez J, Mila-Aloma M, Gispert JD, et al. Novel tau biomarkers phosphorylated at T181, T217 or T231 rise in the initial stages of the preclinical Alzheimer's continuum when only subtle changes in Aβ pathology are detected. EMBO Mol Med. 2020;12(12):e12921. 10.15252/emmm.202012921 PMC7721364 33169916

[10] Mielke MM, Hagen CE, Xu J, Chai X, Vemuri P, Lowe VJ, Airey DC, Knopman DS, Roberts RO, Machulda MM, Jack CR, Petersen RC, Dage JL (2018). Plasma phospho-tau181 increases with Alzheimer's disease clinical severity and is associated with tau- and amyloid-positron emission tomography. Alzheimers Dement.

[11] Meyer PF, Ashton NJ, Karikari TK, Strikwerda-Brown C, Kobe T, Gonneaud J (2022). Plasma p-tau231, p-tau181, PET Biomarkers, and Cognitive Change in Older Adults. Ann Neurol.

[12] Bayoumy S, Verberk IMW, den Dulk B, Hussainali Z, Zwan M, van der Flier WM, Ashton NJ, Zetterberg H, Blennow K, Vanbrabant J, Stoops E, Vanmechelen E, Dage JL, Teunissen CE (2021). Clinical and analytical comparison of six Simoa assays for plasma P-tau isoforms P-tau181, P-tau217, and P-tau231. Alzheimers Res Ther.

[13] Smirnov DS, Ashton NJ, Blennow K, Zetterberg H, Simren J, Lantero-Rodriguez J (2022). Plasma biomarkers for Alzheimer's Disease in relation to neuropathology and cognitive change. Acta Neuropathol.

[14] Ashton NJ, Pascoal TA, Karikari TK, Benedet AL, Lantero-Rodriguez J, Brinkmalm G, Snellman A, Scholl M, Troakes C, Hye A, Gauthier S, Vanmechelen E, Zetterberg H, Rosa-Neto P, Blennow K (2021). Plasma p-tau231: a new biomarker for incipient Alzheimer's disease pathology. Acta Neuropathol.

[15] Trelle AN, Carr VA, Guerin SA, Thieu MK, Jayakumar M, Guo W, et al. Hippocampal and cortical mechanisms at retrieval explain variability in episodic remembering in older adults. Elife. 2020;9:e55335. 10.7554/eLife.55335 PMC7259949 32469308

[16] Trelle AN, Carr VA, Wilson EN, Swarovski MS, Hunt MP, Toueg TN, Tran TT, Channappa D, Corso NK, Thieu MK, Jayakumar M, Nadiadwala A, Guo W, Tanner NJ, Bernstein JD, Litovsky CP, Guerin SA, Khazenzon AM, Harrison MB, Rutt BK, Deutsch GK, Chin FT, Davidzon GA, Hall JN, Sha SJ, Fredericks CA, Andreasson KI, Kerchner GA, Wagner AD, Mormino EC (2021). Association of CSF Biomarkers With Hippocampal-Dependent Memory in Preclinical Alzheimer Disease. Neurology.

[17] Nasreddine ZS, Phillips NA, Bedirian V, Charbonneau S, Whitehead V, Collin I, Cummings JL, Chertkow H (2005). The Montreal Cognitive Assessment, MoCA: a brief screening tool for mild cognitive impairment. J Am Geriatr Soc.

[18] Morris JC (1993). The Clinical Dementia Rating (CDR): current version and scoring rules. Neurology.

[19] Wilson EN, Swarovski MS, Linortner P, Shahid M, Zuckerman AJ, Wang Q, Channappa D, Minhas PS, Mhatre SD, Plowey ED, Quinn JF, Zabetian CP, Tian L, Longo FM, Cholerton B, Montine TJ, Poston KL, Andreasson KI (2020). Soluble TREM2 is elevated in Parkinson’s disease subgroups with elevated CSF tau. Brain.

[20] Lewczuk P, Riederer P, O'Bryant SE, Verbeek MM, Dubois B, Visser PJ, Jellinger KA, Engelborghs S, Ramirez A, Parnetti L, Jack CR, Teunissen CE, Hampel H, Lleo A, Jessen F, Glodzik L, de Leon MJ, Fagan AM, Molinuevo JL, Jansen WJ, Winblad B, Shaw LM, Andreasson U, Otto M, Mollenhauer B, Wiltfang J, Turner MR, Zerr I, Handels R, Thompson AG, Johansson G, Ermann N, Trojanowski JQ, Karaca I, Wagner H, Oeckl P, Waalwijk van Doorn L, Bjerke M, Kapogiannis D, Kuiperij HB, Farotti L, Li Y, Gordon BA, Epelbaum S, SJB V, CJM K, Van Nostrand WE, Minguillon C, Schmitz M, Gallo C, Lopez Mato A, Thibaut F, Lista S, Alcolea D, Zetterberg H, Blennow K, Kornhuber J, Members of the Wfsbp Task Force Working on this Topic: Peter Riederer CGDKALMFT (2018). Cerebrospinal fluid and blood biomarkers for neurodegenerative dementias: An update of the Consensus of the Task Force on Biological Markers in Psychiatry of the World Federation of Societies of Biological Psychiatry. World J Biol Psychiatry.

[21] Feinstein I, Wilson EN, Swarovski MS, Andreasson KI, Angst MS, Greicius MD (2021). Plasma Biomarkers of Tau and Neurodegeneration During Major Cardiac and Noncardiac Surgery. JAMA Neurol.

[22] Vanderstichele H, De Vreese K, Blennow K, Andreasen N, Sindic C, Ivanoiu A, Hampel H, Burger K, Parnetti L, Lanari A, Padovani A, DiLuca M, Blaser M, Olsson AO, Pottel H, Hulstaert F, Vanmechelen E (2006). Analytical performance and clinical utility of the INNOTEST PHOSPHO-TAU181P assay for discrimination between Alzheimer's disease and dementia with Lewy bodies. Clin Chem Lab Med.

[23] Vanmechelen E, Vanderstichele H, Davidsson P, Van Kerschaver E, Van Der Perre B, Sjogren M, Andreasen N, Blennow K (2000). Quantification of tau phosphorylated at threonine 181 in human cerebrospinal fluid: a sandwich ELISA with a synthetic phosphopeptide for standardization. Neurosci Lett.

[24] Goedert M, Jakes R, Crowther RA, Cohen P, Vanmechelen E, Vandermeeren M, Cras P (1994). Epitope mapping of monoclonal antibodies to the paired helical filaments of Alzheimer's disease: identification of phosphorylation sites in tau protein. Biochem J.

[25] Mormino EC, Kluth JT, Madison CM, Rabinovici GD, Baker SL, Miller BL, Koeppe RA, Mathis CA, Weiner MW, Jagust WJ, Alzheimer's Disease Neuroimaging I (2009). Episodic memory loss is related to hippocampal-mediated beta-amyloid deposition in elderly subjects. Brain.

[26] Karikari TK, Pascoal TA, Ashton NJ, Janelidze S, Benedet AL, Rodriguez JL, Chamoun M, Savard M, Kang MS, Therriault J, Scholl M, Massarweh G, Soucy JP, Hoglund K, Brinkmalm G, Mattsson N, Palmqvist S, Gauthier S, Stomrud E, Zetterberg H, Hansson O, Rosa-Neto P, Blennow K (2020). Blood phosphorylated tau 181 as a biomarker for Alzheimer's disease: a diagnostic performance and prediction modelling study using data from four prospective cohorts. Lancet Neurol.

[27] Moscoso A, Grothe MJ, Ashton NJ, Karikari TK, Rodriguez JL, Snellman A, Suarez-Calvet M, Zetterberg H, Blennow K, Scholl M, Alzheimer's Disease Neuroimaging I (2021). Time course of phosphorylated-tau181 in blood across the Alzheimer's disease spectrum. Brain.

[28] O'Connor A, Karikari TK, Poole T, Ashton NJ, Lantero Rodriguez J, Khatun A, Swift I, Heslegrave AJ, Abel E, Chung E, Weston PSJ, Pavisic IM, Ryan NS, Barker S, Rossor MN, Polke JM, Frost C, Mead S, Blennow K, Zetterberg H, Fox NC (2021). Plasma phospho-tau181 in presymptomatic and symptomatic familial Alzheimer's disease: a longitudinal cohort study. Mol Psychiatry.

[29] Therriault J, Benedet AL, Pascoal TA, Lussier FZ, Tissot C, Karikari TK, Ashton NJ, Chamoun M, Bezgin G, Mathotaarachchi S, Gauthier S, Saha-Chaudhuri P, Zetterberg H, Blennow K, Rosa-Neto P, Alzheimer's Disease Neuroimaging I (2021). Association of plasma P-tau181 with memory decline in non-demented adults. Brain Commun.

[30] Moscoso A, Grothe MJ, Ashton NJ, Karikari TK, Lantero Rodriguez J, Snellman A, Suarez-Calvet M, Blennow K, Zetterberg H, Scholl M, Alzheimer's Disease Neuroimaging I (2021). Longitudinal Associations of Blood Phosphorylated Tau181 and Neurofilament Light Chain With Neurodegeneration in Alzheimer Disease. JAMA Neurol.

[31] Pereira JB, Janelidze S, Stomrud E, Palmqvist S, van Westen D, Dage JL, Mattsson-Carlgren N, Hansson O (2021). Plasma markers predict changes in amyloid, tau, atrophy and cognition in non-demented subjects. Brain.

[32] Barthelemy NR, Horie K, Sato C, Bateman RJ. Blood plasma phosphorylated-tau isoforms track CNS change in Alzheimer's disease. J Exp Med. 2020;217(11):e20200861. 10.1084/jem.20200861 PMC7596823 32725127

[33] Palmqvist S, Insel PS, Stomrud E, Janelidze S, Zetterberg H, Brix B, Eichenlaub U, Dage JL, Chai X, Blennow K, Mattsson N, Hansson O (2019). Cerebrospinal fluid and plasma biomarker trajectories with increasing amyloid deposition in Alzheimer's disease. EMBO Mol Med.

[34] Palmqvist S, Janelidze S, Quiroz YT, Zetterberg H, Lopera F, Stomrud E, Su Y, Chen Y, Serrano GE, Leuzy A, Mattsson-Carlgren N, Strandberg O, Smith R, Villegas A, Sepulveda-Falla D, Chai X, Proctor NK, Beach TG, Blennow K, Dage JL, Reiman EM, Hansson O (2020). Discriminative Accuracy of Plasma Phospho-tau217 for Alzheimer Disease vs Other Neurodegenerative Disorders. JAMA.

[35] Leuzy A, Janelidze S, Mattsson-Carlgren N, Palmqvist S, Jacobs D, Cicognola C, Stomrud E, Vanmechelen E, Dage JL, Hansson O (2021). Comparing the Clinical Utility and Diagnostic Performance of CSF P-Tau181, P-Tau217, and P-Tau231 Assays. Neurology.

[36] Ashton NJ, Leuzy A, Karikari TK, Mattsson-Carlgren N, Dodich A, Boccardi M, Corre J, Drzezga A, Nordberg A, Ossenkoppele R, Zetterberg H, Blennow K, Frisoni GB, Garibotto V, Hansson O (2021). The validation status of blood biomarkers of amyloid and phospho-tau assessed with the 5-phase development framework for AD biomarkers. Eur J Nucl Med Mol Imaging.

[37] Boccardi M, Dodich A, Albanese E, Gayet-Ageron A, Festari C, Ashton NJ, Bischof GN, Chiotis K, Leuzy A, Wolters EE, Walter MA, Rabinovici GD, Carrillo M, Drzezga A, Hansson O, Nordberg A, Ossenkoppele R, Villemagne VL, Winblad B, Frisoni GB, Garibotto V (2021). The strategic biomarker roadmap for the validation of Alzheimer's diagnostic biomarkers: methodological update. Eur J Nucl Med Mol Imaging.

[38] Mielke MM, Frank RD, Dage JL, Jeromin A, Ashton NJ, Blennow K, Karikari TK, Vanmechelen E, Zetterberg H, Algeciras-Schimnich A, Knopman DS, Lowe V, Bu G, Vemuri P, Graff-Radford J, Jack CR, Petersen RC (2021). Comparison of Plasma Phosphorylated Tau Species With Amyloid and Tau Positron Emission Tomography, Neurodegeneration, Vascular Pathology, and Cognitive Outcomes. JAMA Neurol.

[39] Hansson O, Edelmayer RM, Boxer AL, Carrillo MC, Mielke MM, Rabinovici GD, et al. The Alzheimer's Association appropriate use recommendations for blood biomarkers in Alzheimer's disease. Alzheimers Dement. 2022:1–18. 10.1002/alz.12756 PMC10087669 35908251

[40] O'Bryant SE, Mielke MM, Rissman RA, Lista S, Vanderstichele H, Zetterberg H, Lewczuk P, Posner H, Hall J, Johnson L, Fong YL, Luthman J, Jeromin A, Batrla-Utermann R, Villarreal A, Britton G, Snyder PJ, Henriksen K, Grammas P, Gupta V, Martins R, Hampel H, Biofluid Based Biomarker Professional Interest A (2017). Blood-based biomarkers in Alzheimer disease: Current state of the science and a novel collaborative paradigm for advancing from discovery to clinic. Alzheimers Dement.

[41] Reddy JS, Jin J, Lincoln SJ, Ho CCG, Crook JE, Wang X, et al. Transcript levels in plasma contribute substantial predictive value as potential Alzheimer's disease biomarkers in African Americans. EBioMedicine. 2022;78:103929. 10.1016/j.ebiom.2022.103929 PMC9044003 35307406

[42] Deniz K, Ho CCG, Malphrus KG, Reddy JS, Nguyen T, Carnwath TP, Crook JE, Lucas JA, Graff-Radford NR, Carrasquillo MM, Ertekin-Taner N (2021). Plasma Biomarkers of Alzheimer's Disease in African Americans. J Alzheimers Dis.

