# Supplementary material for: Performance of a fully-automated Lumipulse plasma phospho-tau181 assay for Alzheimer’s disease
Source: Alzheimers Res Ther. 2022 Nov 12;14:172. doi: 10.1186/s13195-022-01116-2 (PMC9652927; doi:10.1186/s13195-022-01116-2)
Supplement: Supplementary file 1 — Additional file 1: Table S1. Characteristics of Participants Stratified by Diagnosis and APOE Genotype. [file 13195_2022_1116_MOESM1_ESM.docx]

| **Table S1** Characteristics of Participants Stratified by Diagnosis and *APOE* Genotype | | | | |  |
| --- | --- | --- | --- | --- | --- |
| **Diagnosis** | **APOE ε4-/-** | **APOE ε4+/-** | **APOE ε4+/+** | ***p*** |  |
| **Clinically Unimpaired (n =357)** | **n = 257** | **n = 83** | **n = 17** |  |  |
| **Age, y** | 70.9 ± 7.3 | 70.1 ± 7.8 | 70.9 ± 5.9 | ns |  |
| **Sex, female** | 146 (40.9%) | 53 (63.9%) | 11 (64.7%) | ns |  |
| **Plasma P-Tau181, pg/ml** | 1.77 ± 0.8 | 1.83 ± 0.7 | 2.11 ± 0.8*^a^* | 0.0306 |  |
| **Mild Cognitively Impaired (n = 69)** | **n = 39** | **n = 22** | **n = 7** |  |  |
| **Age, y** | 74.4 ± 8.5 | 73.4 ± 8.0 | 72.7 ± 7.5 | ns |  |
| **Sex, female** | 15 (38.5%) | 11 (47.8%) | 4 (57.1%) | ns |  |
| **Plasma P-Tau181, pg/ml** | 1.76 ± 0.6 | 2.33 ± 0.9*^b^* | 2.81 ± 1.3*^b^* | 0.0003 |  |
| **Alzheimer's Disease (n = 47)** | **n = 13** | **n = 26** | **n = 8** |  |  |
| **Age, y** | 69.7 ± 16.3 | 70.5 ± 10.7 | 66.9 ± 6.1 | ns |  |
| **Sex, female** | 4 (30.8%) | 18 (69.2%) | 5 (62.5%) | ns |  |
| **Plasma P-Tau181, pg/ml** | 3.10 ± 0.8 | 3.37 ± 1.4 | 3.82 ± 0.4 | ns |  |
| Abbreviations: AD: Alzheimer's disease; CU: clinically unimpaired; MCI: mild cognitive impairment. Continuous variables expressed as mean ± SD while categorical values are expressed as n (%). a, * vs CU; b, ** vs CU. Data analyzed using one-way ANOVA with Tukey’s post hoc tests for multiple comparisons (age), Chi-square (sex), or by ANCOVA with age and sex included as covariates (Plasma P-Tau181). | | | | |  |
|  |  |  |  |  |  |
|  |  |  |  |  |  |
|  |  |  |  |  |  |
|  |  |  |  |  |  |
|  |  |  |  |  |  |
|  |  |  |  |  |  |
|  |  |  |  |  |  |
|  |  |  |  |  |  |
