# Supplementary material for: Performance of a fully-automated Lumipulse plasma phospho-tau181 assay for Alzheimer’s disease
Source: Alzheimers Res Ther. 2022 Nov 12;14:172. doi: 10.1186/s13195-022-01116-2 (PMC9652927; doi:10.1186/s13195-022-01116-2)
Supplement: Supplementary file 2 — Additional file 2: Table S2. Characteristics of Participants Stratified by Diagnosis and AD CSF Biomarkers. [file 13195_2022_1116_MOESM2_ESM.docx]

| **Table S2** Characteristics of Participants Stratified by Diagnosis and AD CSF Biomarkers | | | | | | |  |
| --- | --- | --- | --- | --- | --- | --- | --- |
|  | **Aβ- CU** | **Aβ+ CU** | **Aβ- MCI** | **Aβ+ MCI** | **Aβ+ AD** | ***p*** |  |
| **n** | 134 | 55 | 10 | 20 | 17 |  |  |
| **Age, y** | 67.4 ± 6.9 | 69.9 ± 7.7 | 69.8 ± 10.6 | 72.0 ± 5.9 | 66.0 ± 9.4 | ns |  |
| **Sex, female** | 75 (60.1%) | 33 (60.0%) | 5 (50.0%) | 5 (25.0%) | 15 (88.2%) | 0.004 |  |
| **Plasma P-Tau181, pg/ml** | 1.48 ± 0.39 | 1.90 ± 0.62^a^ | 1.59 ± 0.44 | 2.03 ± 0.48^b^ | 3.83 ± 1.55^c^ | <0.0001 |  |
| Abbreviations: AD: Alzheimer's disease; CU: clinically unimpaired; MCI: mild cognitive impairment. Continuous variables expressed as mean ± SD while categorical values are expressed as n (%). a, *** vs Aβ- CU; b, ** vs Aβ- CU.; c, *** vs all groups. Data analyzed using one-way ANOVA with Tukey’s post hoc tests for multiple comparisons (age), Chi-square (sex), or by ANCOVA with age and sex included as covariates (Plasma P-Tau181). | | | | | | |  |
|  |  |  |  |  |  |  |  |
|  |  |  |  |  |  |  |  |
|  |  |  |  |  |  |  |  |
|  |  |  |  |  |  |  |  |
|  |  |  |  |  |  |  |  |
|  |  |  |  |  |  |  |  |
|  |  |  |  |  |  |  |  |
|  |  |  |  |  |  |  |  |
