# Supplementary material for: Performance of a fully-automated Lumipulse plasma phospho-tau181 assay for Alzheimer’s disease
Source: Alzheimers Res Ther. 2022 Nov 12;14:172. doi: 10.1186/s13195-022-01116-2 (PMC9652927; doi:10.1186/s13195-022-01116-2)
Supplement: Supplementary file 3 — Additional file 3: Table S3. Betas (standard errors) and p values within and between clinical groups for models examining longitudinal change in plasma p-tau181. [file 13195_2022_1116_MOESM3_ESM.docx]

| **Table S3** Betas (standard errors) and p values within and between clinical groups for models examining longitudinal change in plasma p-tau181 | | |
| --- | --- | --- |
|  | **B (SE)** | ***p*** |
| **Change vs. 0 per clinical group** | | |
| **CU** | +0.090 (0.038) | 0.016 |
| **MCI** | +0.128 (0.080) | 0.107 |
| **AD** | +0.355 (0.114) | 0.002 |
| **Comparison of change between clinical groups** | | |
| **MCI vs. CU** | +0.038 (0.088) | 0.667 |
| **AD vs. CU** | +0.265 (0.120) | 0.027 |
| **AD vs. MCI** | +0.227 (0.139) | 0.102 |
