# Supplementary material for: Performance of a fully-automated Lumipulse plasma phospho-tau181 assay for Alzheimer’s disease
Source: Alzheimers Res Ther. 2022 Nov 12;14:172. doi: 10.1186/s13195-022-01116-2 (PMC9652927; doi:10.1186/s13195-022-01116-2)
Supplement: Supplementary file 4 — Additional file 4: Table S4. Betas (standard errors) and p values for models examining plasma p-tau181 and cross-sectional cognition and function. [file 13195_2022_1116_MOESM4_ESM.docx]

| **Table S4** Betas (standard errors) and p values for models examining plasma p-tau181 and cross-sectional cognition and function | | |
| --- | --- | --- |
|  | **B (SE)** | ***p*** |
| **Plasma P-Tau181 Predicting Global Cognition** | | |
| **Associations per clinical group** | | |
| **CU** | -0.095 (0.287) | 0.742 |
| **MCI** | -0.167 (0.499) | 0.738 |
| **AD** | -1.661 (0.458) | < 0.001 |
| **Comparison of associations between clinical groups** | | |
| **MCI vs. CU** | -0.073 (0.573) | 0.899 |
| **AD vs. CU** | -1.566 (0.547) | 0.006 |
| **AD vs. MCI** | -1.493 (0.676) | 0.028 |
| **Plasma P-Tau181 Predicting Function** | | |
| **Associations per clinical group** | | |
| **CU** | +0.032 (0.106) | 0.765 |
| **MCI** | +0.449 (0.225) | 0.047 |
| **AD** | +1.336 (0.196) | <0.001 |
| **Comparison of associations between clinical groups** | | |
| **MCI vs. CU** | +0.418 (0.248) | 0.094 |
| **AD vs. CU** | +1.304 (0.225) | <0.001 |
| **AD vs. MCI** | +0.886 (0.299) | 0.003 |
