# Supplementary material for: Performance of a fully-automated Lumipulse plasma phospho-tau181 assay for Alzheimer’s disease
Source: Alzheimers Res Ther. 2022 Nov 12;14:172. doi: 10.1186/s13195-022-01116-2 (PMC9652927; doi:10.1186/s13195-022-01116-2)
Supplement: Supplementary file 5 — Additional file 5: Table S5. Betas (standard errors) and p values for models examining plasma p-tau181 and longitudinal change in global cognition and function. [file 13195_2022_1116_MOESM5_ESM.docx]

| **Table S5** Betas (standard errors) and p values for models examining plasma p-tau181 and longitudinal change in global cognition and function | | |
| --- | --- | --- |
|  | **B (SE)** | ***p*** |
| **Plasma P-Tau181 Predicting Change in Global Cognition** | | |
| **Associations per clinical group** | | |
| **CU** | -0.007 (0.094) | 0.943 |
| **MCI** | -0.748 (0.178) | <0.001 |
| **AD** | -0.498 (0.282) | 0.078 |
| **Comparison of associations between clinical groups** | | |
| **MCI vs. CU** | -0.742 (0.203) | <0.001 |
| **AD vs. CU** | -0.491 (0.304) | 0.107 |
| **AD vs. MCI** | -0.250 (0.332) | 0.452 |
| **Plasma P-Tau181 Predicting Change in Function** | | |
| **Associations per clinical group** | | |
| **CU** | -0.002 (0.026) | 0.953 |
| **MCI** | +0.049 (0.045) | 0.279 |
| **AD** | +0.300 (0.060) | <0.001 |
| **Comparison of associations between clinical groups** | | |
| **MCI vs. CU** | +0.050 (0.052) | 0.34 |
| **AD vs. CU** | +0.302 (0.068) | <0.001 |
| **AD vs. MCI** | +0.252 (0.074) | <0.001 |
